# Focus on Women with Hemophilia and Carriers: How to Address All the Existing Gaps in Diagnosis and Care

**DOI:** 10.3390/jcm15155885

**Published:** 2026-07-28

**Authors:** Paola Ranalli, Lucia Di Re, Francesco Guardalupi, Daniela Bruno, Mauro Di Ianni

**Affiliations:** 1 Department of Medicine and Aging Sciences, University of Chieti-Pescara, 66100 Chieti, Italy; 2 Hematology Unit, Pescara Hospital, 65124 Pescara, Italy; 3 Transfusion Medicine Unit, Hemostasis Laboratory, S. Spirito Hospital, 65124 Pescara, Italy

**Keywords:** bleeding phenotype, equity, hemophilia, multidisciplinary care, women

## Abstract

**Background/Objectives:** Research on hemophilia carriers and women and girls with hemophilia (WGH) has progressed more slowly than studies involving male patients. In recent years, however, attention to female carriers and affected women has increased substantially. Emerging evidence shows that carriers may experience significant bleeding symptoms regardless of their coagulation factor levels, with a notable impact on quality of life. Despite this, gender-based disparities continue to delay diagnosis and limit access to appropriate clinical management and treatment. Women also face a considerable psychosocial burden—not only as carriers or affected individuals but also as mothers or just caregivers or partners of male patients. Closing this gap requires proactive screening, regular pedigree updates, and, above all, improved education among healthcare professionals to ensure equitable care for all. **Methods:** A literature review was conducted to summarize current data and advances in this field while highlighting areas that remain unclear and require more effective management strategies. **Results:** The findings emphasize the importance of a more inclusive approach to hemophilia care, addressing diagnostic, therapeutic and psychosocial challenges in a systematic way. **Conclusions:** This review underscores the need for fair recognition and comprehensive care across the full spectrum of female experiences with hemophilia.

## 1. Introduction

Hemophilia is an X-linked recessive bleeding disorder caused by a deficiency of coagulation factor VIII (FVIII; hemophilia A) or factor IX (FIX; hemophilia B) [1]. Owing to its X-linked inheritance, hemophilia has traditionally been regarded as a predominantly male condition, leading to the under-recognition of bleeding symptoms in female carriers, who were historically considered clinically unaffected. This longstanding misconception has contributed to limited clinical attention and research involving women with hemophilia and hemophilia carriers [2,3,4].

Without treatment, individuals with severe hemophilia—defined by FVIII or FIX levels below 1 U/dL—are at high risk of spontaneous musculoskeletal hemorrhages, which represent approximately 80% of all bleeding episodes [5]. Life-threatening events, including intracranial hemorrhage, may also occur [6]. Recurrent joint bleeding (hemarthrosis), particularly in the ankles, elbows, and knees, can result in progressive and irreversible joint damage (hemophilic arthropathy) if not adequately managed [6].

Advances in biomolecular diagnostic techniques by the late 20th century substantially improved the identification of hemophilia carriers [7]. However, it was only in 2021 that the Scientific and Standardization Committee of the International Society on Thrombosis and Haemostasis (ISTH) introduced the term “women and girls with hemophilia” (WGH), which remains in use today [8].

A prospective study published in 2021 involving female sisters of patients with hemophilia A or B reported that nearly half of participants (46%) had FVIII or FIX levels within the range of mild hemophilia [9]. Notably, abnormal bleeding is not limited to carriers with reduced coagulation factor levels [8]. In the same cohort, female sisters exhibited an increased bleeding tendency compared with the general population, regardless of whether their factor levels were within the normal range [9].

In recent decades, the increased availability of therapeutic options—including both replacement and non-replacement agents—has enabled higher levels of bleed protection and reduced treatment burden for male patients receiving prophylaxis in high-resource settings [6]. Recent studies have demonstrated that trough levels ≥ 15% are required to protect patients from all joint bleeding, thereby substantially reducing the risk of arthropathy and improving quality of life [10,11,12].

Hemophilia significantly affects daily functioning across all ages and severities, particularly limiting physical activity and social participation. This impact is not strictly correlated with disease severity, as individuals with moderate hemophilia have been reported to experience lower quality of life than those with severe disease [13].

These insights have increased awareness of bleeding risk in non-severe patients, including females, and have prompted discussion regarding the potential need for prophylaxis in this population [8], particularly among individuals with a pronounced bleeding phenotype or those at elevated risk due to lifestyle or sports participation [14,15].

A comprehensive literature search was conducted between January 2026 and April 2026 to identify relevant studies published. The electronic database consulted was PubMed. The search strategy combined free-text keywords related to the core themes of this review, including “women and hereditary bleeding disorders,” “women and hemophilia,” “women with hemophilia and bleeding,” and more specific combinations such as “women with hemophilia and anemia,” “women with hemophilia and gynecological bleeding,” “women with hemophilia and arthropathy,” and “women with hemophilia and psychological burden.” The search was restricted to articles published in English, and the reference lists of retrieved studies were manually screened to identify additional eligible publications.

## 2. Diagnosis and Laboratory Issues

Women with hemophilia experience substantial delays in diagnosis, with the median time interval between symptom onset and diagnosis averaging 8 years (range: 6 months–30 years), reflecting a critical gap in the recognition and testing of hemophilia in female populations [16]. Reported data indicate a median age of 27 years at diagnosis, with a broad range that reflects the heterogeneity of clinical presentation and the timing of evaluation [17]. Many cases remain undiagnosed for years despite clear bleeding symptoms [18].

**Figure 1:**
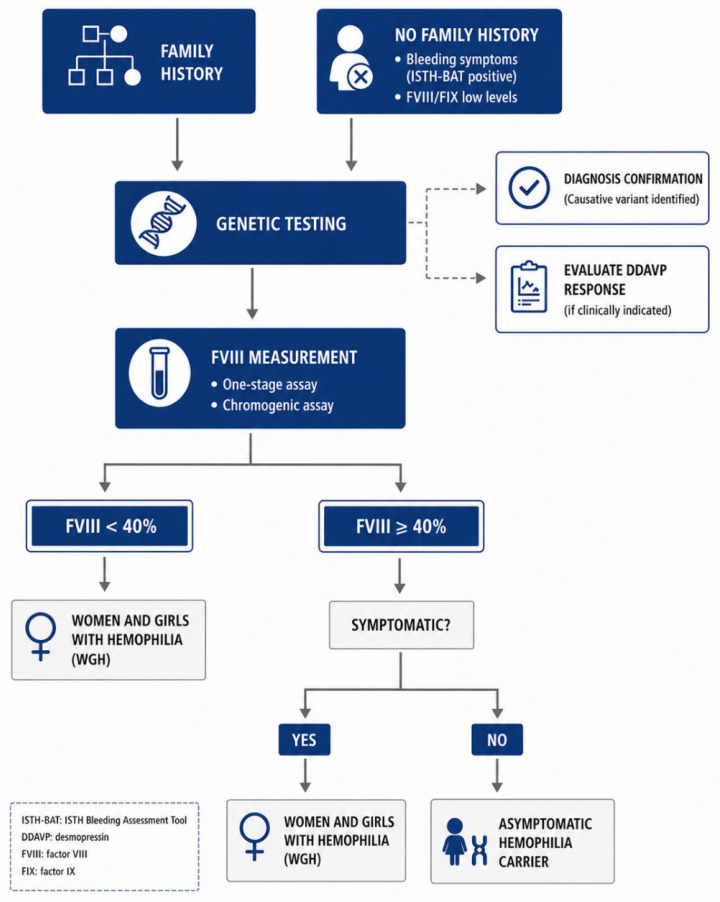
Diagnostic pathway in women suspected of having hemophilia A or B or carriers, with or without a family history of the condition.

Laboratory diagnosis of women with hemophilia and carriers is complex and requires more than factor-level testing. Figure 1 depicts a suitable diagnostic pathway for carriers and affected females.

Beyond cultural barriers, diagnostic complexity also stems from inconsistencies between the laboratory assays used to measure coagulation factor levels. Among hemophilia A carriers, 23% show factor VIII activity (FVIII:C) levels below 40 IU/dL when assessed using a one-stage assay, whereas 41% demonstrate discrepant results when evaluated with a chromogenic assay. Therefore, a robust laboratory evaluation—including both assay types—is essential for accurate phenotyping in all suspected cases [19].

As previously reported, FVIII levels in females do not reliably predict bleeding symptoms. Because von Willebrand factor (vWF) levels increase with age, FVIII levels also rise in older women, which may explain why FVIII values often do not correlate with ISTH-Bleeding Assessment Tool (BAT) scores [20]. This age-related physiological shift further complicates diagnostic evaluation in women who undergo testing later in life, as changes in coagulation factor levels may obscure the relationship between laboratory findings and clinical bleeding manifestations. Additional factors—including hormonal fluctuations, stress, and pregnancy—are also known to influence FVIII levels, potentially shifting values around clinically relevant hemostatic thresholds [21,22,23].

Conversely, modifiers identified in male patients with severe hemophilia A as being associated with lower FVIII levels—such as low vWF levels and blood group O—do not adequately explain FVIII values in females, a phenomenon already observed in males with non-severe hemophilia [24].

**Figure 2:**
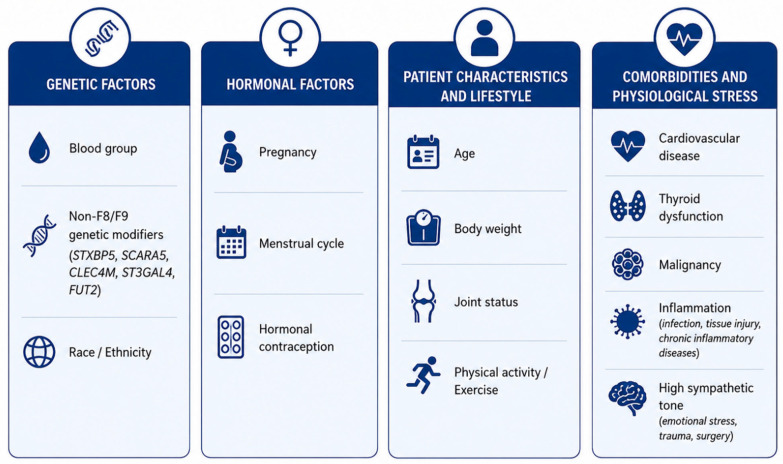
List of sex and non-sex-specific factors influencing FVIII and vWF circulating levels in women who are affected or carriers.

Figure 2 summarizes the factors influencing FVIII and vWF levels in women with hemophilia and in hemophilia carriers.

Standard coagulation tests—including PT, aPTT, and platelet count—do not correlate with bleeding phenotype or bleeding assessment tool scores and therefore should not be used as surrogate screening tests in diagnostic or preoperative evaluations [25]. This dissociation between laboratory parameters and clinical bleeding is not unique to women with hemophilia; it is a well-recognized challenge across nearly all rare bleeding disorders (RBDs) [26].

Traditional bleeding assessment tools, particularly the ISTH-BAT, provide a structured measure of bleeding severity in women with hemophilia and carriers [16]. However, ISTH-BAT scores show poor correlations with coagulation factor levels. Among hemophilia B carriers, factor IX activity and age together accounted for only 30.9% of the variability in bleeding scores [27]. In a recently published study, approximately 50% of female participants with baseline FVIII > 40% exhibited abnormal BAT results [28]. Similarly, a multicenter Italian study found no significant association between bleeding events and factor levels (*p* > 0.05), although an inverse trend between ISTH-BAT scores and factor activity was noted (*p* = 0.08) [17].

These findings highlight that bleeding scores reflect complex hemostatic influences extending beyond factor activity alone and that they more reliably capture the expected clinical phenotype. Consequently, systematic assessment of bleeding history remains essential for all women with hemophilia or carrier status, regardless of factor levels [27]. Emerging diagnostic approaches—such as assays evaluating the response to hemostatic stress (e.g., desmopressin challenge), global coagulation assays, and clot waveform analysis—may offer additional prognostic value, but they are not yet incorporated into routine clinical practice [29]. Until more refined predictive models are validated, standardized bleeding assessment tools remain the most dependable method for identifying women at risk of bleeding complications and should be applied consistently in this population [30].

Statistical models are needed for suspected carriers or females with hemophilia to better predict abnormal bleeding phenotypes and the potential need for prophylaxis. A practical, low-cost prediction model combining the ISTH-BAT, factor levels, and age has been developed to identify and classify female hemophilia carriers, achieving 94.7% accuracy. This approach is particularly valuable in resource-limited settings, enabling early identification of at-risk women before pursuing more expensive genetic testing [31]. Another model incorporating skewed X-chromosome inactivation (XCI) and FVIII antigen/activity as interaction terms significantly improved logistic regression performance compared with FVIII alone, showing promising predictive capability [28].

Machine learning approaches may ultimately prove highly effective in predicting bleeding risk by evaluating multiple factors simultaneously and identifying complex patterns and previously unrecognized interactions [32].

## 3. Nomenclature

Historically, women with hemophilia were described as *carriers*, independently of the bleeding phenotype and coagulation factor observed at baseline; this term is rooted in the outdated assumption that hemophilia was an exclusively male disorder and that affected women served only as genetic transmitters, without clinical involvement. This nomenclature update mirrors a more accurate understanding of hemophilia as a condition that affects individuals of all sexes [33]. Abnormal BAT scores and remarkable bleeding phenotypes are observed in up to 50% of females with FVIII:C > 40%; thus, they appear to be clinically similar to females with FVIII:C < 40% and mild hemophilia A (HA) males [28].

The revised terminology seems to more accurately reflect the clinical needs of this population, who frequently experience clinically significant bleeding; supports improved access to appropriate medical care; and promotes equitable inclusion in clinical research [18].

The updated ISTH nomenclature standardizes the diagnosis of women with hemophilia by incorporating both factor levels and bleeding symptoms. According to the International Society on Thrombosis and Haemostasis/Scientific and Standardization Committee (ISTH/SSC) women, five categories—mild, moderate, and severe hemophilia, plus symptomatic and asymptomatic carriers are recognized, based on FVIII/IX thresholds and clinical phenotype. Women with or without hemophilia family history, who show pathogenic variants in *F8* or *F9* and exhibit reduced factor VIII or factor IX levels—particularly below 40 IU/dL—are now classified as Women and Girls with Hemophilia (WGH). Women carriers of pathogenic variants showing a baseline FVIII or FIX levels < 40% are still called “carriers”, with a further classification into “symptomatic” and “asymptomatic carriers” depending on bleeding phenotype [8]. According to a recent study almost half sisters of hemophilia male patients enrolled and previously considered just “carriers” show factor levels in the range of mild hemophilia actually [9].

## 4. Genetics in Women

It is estimated that, for every man diagnosed with hemophilia, approximately 1.6 women are identified as carriers [19]. Carrier status or disease is frequently unrecognized until a male family member receives a diagnosis or during pregnancy [34]. As management strategies for this subgroup have advanced, the approach to genetic testing has evolved accordingly. Whereas testing was previously performed primarily before pregnancy to assess the risk of having an affected male fetus, it is now incorporated into routine diagnostic pathways for women and ideally conducted early in life, irrespective of pregnancy status [18].

Genetic sequencing to identify *F8* gene mutations is increasingly utilized, as specific variants may indicate a higher bleeding risk and influence desmopressin responsiveness in females with hemophilia A [35]. However, in contrast to affected males, *F8* and *F9* pathogenic variants in females did not show a significant association with bleeding phenotype in a recent single-center cross-sectional study, with highly symptomatic individuals represented across all mutation types. In hemophilia B carriers (HBCs), insertions and deletions were more common among those without bleeding symptoms, whereas missense and nonsense mutations were more frequently observed in those with a pronounced bleeding tendency. Among hemophilia A carriers (HACs), intron 22 inversion was the most prevalent variant across all bleeding severity groups, including those with milder phenotypes [36].

Genotype–phenotype correlations in female carriers of *F9* mutations may be further complicated by competitive binding between residual wild-type FIX and mutant FIX at extravascular sites [37]. The mechanisms linking a specific gene variant to the clinical phenotype in female carriers remain incompletely understood. Extremely low factor levels rarely result from biallelic mutations, which would lead to full expression of hemophilia severity. Skewed X-chromosome inactivation (XCI) has been proposed as a mechanism underlying markedly reduced FVIII levels in some carriers [38]. Nonetheless, increased bleeding tendency is also observed in hemophilia A carriers with normal FVIII levels and random XCI patterns, suggesting that genetic contributors beyond simple factor deficiency influence bleeding risk [35].

## 5. Type of Bleeding

**Figure 3:**
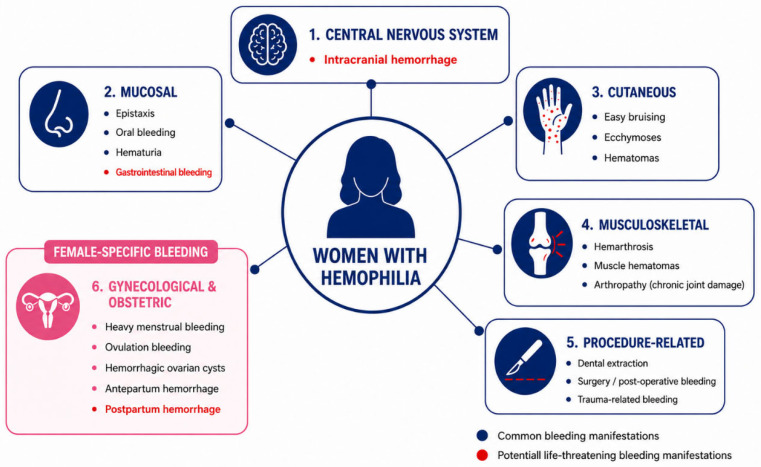
Multisystem bleeding manifestations in women with hemophilia.

Excluding gender-related bleeding, women with hemophilia and carriers experience a broad spectrum of mucocutaneous and systemic bleeding manifestations that are frequently overlooked or underappreciated. These non-gynecological symptoms highlight that bleeding in women with hemophilia involves multiple organ systems and that comprehensive bleeding risk assessment is required for all invasive procedures and traumatic events—not solely those related to reproductive health [39] (Figure 3).

Women with mild factor deficiency and men with mild hemophilia exhibit similar hemorrhagic profiles, with comparable rates of mucocutaneous, muscular, and post-surgical bleeding. This occurs despite women presenting with higher mean factor levels at diagnosis (29% vs. 19%, *p* < 0.0001), receiving a later diagnosis (28.3 vs. 16.4 years, *p* < 0.02), and being treated less frequently with factor concentrates [40]. The most common non-gynecological bleeding manifestations include easy bruising and cutaneous bleeding (reported in 53–60% of women with hemophilia); oral cavity and gingival bleeding (reported in 56.6% of carriers); and epistaxis, which occurs in up to 25% of affected women [39,41].

Post-surgical and post-traumatic bleeding represent particularly significant complications, with major surgical bleeding documented in 29% of women with hemophilia A [41]. More severe bleeding events include joint and muscle hemorrhages, challenging the traditional perception that women with hemophilia have a joint-sparing phenotype. Hemarthroses have been reported in 4–19% of carriers, and subclinical joint bleeding can be detected by ultrasound, even in asymptomatic women [30]. Joint bleeding occurs across all ages and becomes the predominant bleeding site in carriers over 50 years of age [42].

In both hemophilia A and B carriers, bleeding events occur most frequently in adults aged 13–49 years, with significantly higher rates than in pediatric or older populations (*p* < 0.001). In hemophilia B carriers, trauma is the leading trigger across all age groups, whereas in hemophilia A carriers, it predominates only in children aged 0–12 years (55%) [39]. Clinically relevant bleeding also occurs in carriers with normal factor levels; among hemophilia A carriers with FVIII:C > 40%, hemarthrosis was reported in 19% in a prospective study, consistent with earlier findings of approximately 14% [3,43].

### 5.1. Gynecological Bleeding

Gynecological bleeding represents one of the most significant and debilitating clinical manifestations of hemophilia in women and is often the primary symptom leading to diagnosis. Existing research in this population has largely focused on heavy menstrual bleeding during regular menses and postpartum hemorrhage as the predominant bleeding concerns [44]. Heavy menstrual bleeding (HMB) is the most common gynecological manifestation among hemophilia carriers, affecting 64.3% of women with hemophilia A—far exceeding the approximately 10% prevalence observed in non-carrier controls [45]. Objective assessments using the Pictorial Blood Loss Assessment Chart (PBAC) further highlight this burden: nearly all adult hemophilia B carriers report PBAC scores > 100, consistent with excessive menstrual blood loss, and 83.3% of female relatives of individuals with hemophilia A experience menorrhagia [37,46].

Despite its high prevalence, HMB in women with hemophilia is frequently not managed by specialists in bleeding disorders and is often redirected to primary care or gynecology. Paradoxically, these symptoms may be trivialized or normalized, particularly when a family history of HMB is present [47].

An integrated, multidisciplinary approach combining targeted hemostatic therapy, hormonal regulation, and iron repletion offers the most effective strategy for managing HMB in women with hemophilia [48]. Therapeutic options include antifibrinolytic agents such as tranexamic acid, hormonal treatments—most notably combined oral contraceptives and progestin-based regimens—and factor replacement therapy when clotting factor levels are reduced [49]. The levonorgestrel-releasing intrauterine system (LNG-IUS) has proven to be a highly effective intervention, with reported reductions in menstrual blood loss of up to 90% [50].

Ovulation-related or mid-cycle bleeding has not been well characterized as a distinct clinical manifestation in women with hemophilia or hemophilia carriers. Hemophilia-specific studies have not systematically evaluated ovulatory or mid-cycle bleeding in terms of prevalence, severity, or management. As a result, ovulation-associated bleeding likely represents an underexplored component of the bleeding phenotype in women with hemophilia and warrants further investigation. Although intermenstrual bleeding is described within the broader literature on abnormal uterine bleeding and breakthrough bleeding is recognized as a potential adverse effect of certain hormonal therapies, these phenomena have not been specifically studied in the context of hemophilia [51].

Pregnancy introduces unique management challenges for women with hemophilia. Although many carriers have normal or near-normal baseline factor levels, gestation is typically associated with an increase in FVIII and FIX concentrations; however, this rise may be insufficient to fully mitigate bleeding risk [17]. Preconception and prenatal care should include assessment of clotting factor levels in the third trimester, as levels above 50% are generally required to support safe delivery and the use of neuraxial anesthesia [52]. Instrumental delivery is not recommended because of the increased risk of maternal bleeding; it is also contraindicated when a male fetus is affected, given the heightened risk of intracranial hemorrhage [53]. Optimal peripartum care requires coordinated involvement of a multidisciplinary team, including hematologists, obstetricians, pediatric hematologists, anesthesiologists, and maternal–fetal medicine specialists [54].

Postpartum hemorrhage (PPH) occurs in approximately one-third of pregnancies among women who are carriers of hemophilia A or B, with severe PPH reported in 10–17.6% of deliveries—substantially higher than the rates observed in the general population [55,56]. In a multicenter study involving 94 women, 174 pregnancies were documented, resulting in 95 vaginal deliveries and 59 cesarean sections [17]. Bleeding complications occurred in 6.7% of pregnancies, while delivery-related and postpartum hemorrhages were reported in 12.9% of cases [17]. Notably, this elevated risk persisted even among women who received prophylactic clotting factor replacement at the time of delivery, as PPH rates did not significantly differ between those with factor levels < 80 IU/dL who received prophylaxis and those with higher baseline levels who did not [56].

Additional gynecological complications—including antepartum hemorrhage, miscarriage, and hemorrhagic ovarian cysts—further contribute to the substantial morbidity experienced by women with hemophilia [57].

### 5.2. Joint Bleeding and Hemophilic Arthropathy

Women with hemophilia and hemophilia carriers experience substantial musculoskeletal morbidity. Importantly, joint complications are reported across the full spectrum of factor activity levels. Individuals with factor levels below 40% do not exhibit a higher prevalence of joint symptoms than those whose levels exceed 40%, indicating that factor activity alone is an insufficient predictor of musculoskeletal involvement [58]. Nevertheless, long-term data from the Swedish Registry over a 22-year period demonstrated that women with factor levels < 40% have not only a higher risk of joint disease but also a more frequent need for joint surgery than carriers with normal factor levels and women in the general population [59].

In a recent study, 53.5% of hemophilia carriers reported musculoskeletal symptoms—most commonly joint pain (50%) and joint swelling (28.6%)—compared with only 3.3% of age-matched controls [60]. Joint involvement represents a major clinical concern in this population, as subclinical hemarthroses may occur and progressively lead to irreversible joint damage [61]. Functional musculoskeletal alterations also appear more pronounced in women: comparative analyses show significantly higher mean Hemophilia Joint Health Score (HJHS) in female carriers than in controls (4.7 vs. 1.5, *p* < 0.001), indicating poorer joint status [60]. Female hemophilia A carriers have additionally been shown to have a reduced overall joint range of motion, most prominently affecting the knees [62]. Overweight or obese women (BMI > 25 kg/m^2^) with hemophilia also exhibit a significantly increased risk of joint bleeding compared with those of normal weight (25% vs. 0%, *p* = 0.03) [60].

Timely detection of joint involvement is essential to prevent progression toward irreversible musculoskeletal damage [61]. Women with hemophilia require structured follow-up and individualized treatment strategies to prevent and manage joint complications, recognizing that their bleeding phenotype often does not align with traditional factor-level classifications. Optimal musculoskeletal care relies on coordinated, multidisciplinary management involving hematologists, orthopedic specialists, and rehabilitation professionals [63].

Previous imaging studies using magnetic resonance imaging (MRI) and ultrasound have demonstrated early structural joint abnormalities in clinically asymptomatic joints of males with hemophilia [64,65]. This raised the hypothesis that factor levels in hemophilia carriers may be sufficiently low to contribute to joint bleeding in some individuals and to arthropathy as a long-term complication. Subclinical joint bleeding in carriers has indeed been shown to lead to joint damage, as demonstrated by MRI findings. In a prospective study by Gilbert, the ankles exhibited the highest percentage of joint pathology: among 12 ankles examined, 58% (7/12) showed hemosiderin deposition, and 17% (2/12) had an osteochondral cyst. The hips showed the least pathology, with only 2 out of 16 MRIs revealing an osteochondral cyst. Both elbow MRIs demonstrated small hemosiderin deposits, and hemosiderin deposition was observed in two out of six knee MRIs [66].

The role of ultrasound in detecting joint involvement in hemophilia carriers remains debated. Although ultrasound can identify joint abnormalities, its utility is largely limited to detecting clinical synovitis. This limitation creates a diagnostic gap, as early joint changes that precede synovitis may remain undetected with standard imaging approaches. To address this, the Hemophilia Early Arthropathy Detection with Ultrasound (HEAD-US) protocol is increasingly applied to evaluate joint status in women; however, longitudinal data supporting its use specifically in female hemophilia populations remain limited [59,67].

In a recent study, HEAD-US scores of women with factor levels within the mild hemophilia range did not differ significantly from those of carriers with normal factor activity. Similarly, the prevalence of joint pain (*p* = 0.36) and joint swelling (*p* = 0.15) was comparable between the two groups [45].

## 6. Osteoporosis

Research specifically addressing osteoporosis in women with hemophilia and hemophilia carriers remains scarce. Nonetheless, the coexistence of hemophilic arthropathy and osteoporosis has gained increasing attention due to its substantial impact on functional outcomes and quality of life [68]. Hemophilia is frequently associated with reduced bone mass and decreased bone mineral density [69]. Although osteoporosis is recognized in individuals of both sexes with hemophilia, it remains a critical yet sometimes underappreciated aspect of long-term management.

Women with hemophilia and carriers may face unique bone health challenges arising from multiple interrelated mechanisms. Chronic blood loss from heavy menstrual bleeding gradually depletes iron stores and may lead to anemia. Both iron deficiency and anemia impose sustained pressure on the hematopoietic system, a physiological strain linked to accelerated bone loss—a relationship already demonstrated in women with rheumatoid arthritis. Joint bleeds and hemophilic arthropathy may also trigger persistent low-grade inflammation, further compromising skeletal integrity. Reduced mobility, often secondary to joint damage, adds another layer of risk by limiting the mechanical stimuli essential for maintaining bone strength. Additionally, sex-specific differences in bone biology must be considered: women naturally have a lower baseline bone mineral density, and the menopausal transition represents a critical period during which bone loss accelerates markedly [70].

Despite these risks, specific recommendations for diagnosing and treating osteoporosis in women with bleeding disorders are lacking. Hemophilia carriers and women with von Willebrand disease show higher rates of osteoporosis (6.4% vs. 3.5% in controls) and fractures (11.7% vs. 5.8%), corresponding to a relative risk of 1.8 for osteoporosis and 2.0 for fractures [71]. Women more commonly experience vertebral and wrist fractures, whereas men with bleeding disorders tend to develop hip fractures at older ages [70].

Assessment of bone health in women with hemophilia follows general population guidelines but requires heightened clinical vigilance. Dual-energy X-ray absorptiometry (DXA) remains the gold standard for evaluating bone mineral density, with osteoporosis defined by T-scores ≤ −2.5 at the lumbar spine, total hip, or femoral neck. In premenopausal women, Z-scores—adjusted for ethnicity or race—are preferred, with values ≤ −2.0 indicating abnormally low bone density [72].

De la Corte Rodríguez et al. proposed a risk-stratified therapeutic approach for osteoporosis in women with hemophilia. For individuals with moderate fracture risk (typically up to 65–70 years of age), selective estrogen receptor modulators are recommended. In cases of high fracture risk, bisphosphonates or denosumab are suggested as first-line therapies, with parenteral formulations (intravenous zoledronate or subcutaneous denosumab) preferred for women with a history of gastrointestinal bleeding. For those at very high fracture risk, anabolic agents such as teriparatide or abaloparatide, as well as dual-action therapies like romosozumab, are advised; these latter options warrant particular consideration because they actively stimulate bone formation rather than merely slowing bone resorption [73,74]. Finally, prevention plays a central role in maintaining bone health, and multidisciplinary teams managing women with bleeding disorders should focus on making weight-bearing recommendations; implementing exercise-based interventions tailored to individual bleeding risk; recommending dietary measures for an adequate intake of calcium, vitamin D, and proteins; promoting smoking cessation; limiting alcohol consumption; and adopting safe walking practices to reduce fall-related injuries [75].

## 7. Anemia

The chronic nature of bleeding in women with hemophilia leads to secondary complications—most notably iron deficiency anemia—that substantially affect both physical health and psychological well-being. Rates of iron deficiency are remarkably high: in one study, 93% of hemophilia B carriers had ferritin levels below 50 ng/mL, indicating depleted iron stores [27]. For this reason, the Medical and Scientific Advisory Council (MASAC) recommends routine screening for iron deficiency in all women and girls with inherited bleeding disorders, even when hemoglobin levels are within the normal range [76].

More broadly, iron deficiency anemia affects approximately 45% of women with inherited bleeding disorders, driven largely by heavy menstrual bleeding (HMB), the most common bleeding manifestation in women with hemophilia [41]. Among women with inherited bleeding disorders, HMB is associated with a 1.92-fold increase in the odds of anemia compared with women without bleeding disorders [77].

The consequences of iron deficiency anemia extend well beyond hematologic impairment. Iron deficiency negatively affects cognitive performance, reduces physical work capacity, and diminishes overall quality of life—effects that are frequently overlooked and therefore remain untreated [78]. The physical fatigue and reduced exercise tolerance associated with iron deficiency can limit engagement in protective health behaviors, creating a bidirectional cycle in which decreased physical activity contributes to mood disturbances and vulnerability to depression, further reducing motivation for self-care. In addition, iron deficiency anemia exacerbates the clinical burden of bleeding, as anemic women have reduced oxygen-carrying capacity and are consequently at greater risk of deterioration during significant bleeding episodes.

Despite the high prevalence of iron deficiency among women with hemophilia, screening and management practices remain inconsistent [78], with substantial gaps in routine assessment and iron replacement protocols across hemophilia treatment centers. Management typically involves oral iron supplementation—most commonly ferrous sulfate—although intravenous formulations such as ferric carboxymaltose have demonstrated superior efficacy and tolerability, particularly in women with ongoing heavy menstrual bleeding [79].

The combination of chronic bleeding, iron depletion, and inadequate iron repletion results in many women with hemophilia experiencing the compounded burden of anemia alongside their primary bleeding disorder, substantially diminishing their health status, functional capacity, and overall quality of life [44].

## 8. Treatment

Hemophilia A carriers are more frequently treated with non-factor hemostatic therapies—including desmopressin—than hemophilia B carriers [42]. Antifibrinolytic agents also play a central role in reducing and treating bleeding complications, offering an accessible and often self-managed therapeutic option [80]. Their use has increased over the past decade [42]. Tranexamic acid (TXA) remains the first-line adjunctive therapy for women with inherited bleeding disorders, including hemophilia carriers, to reduce perioperative bleeding and limit the need for factor replacement during surgical or dental procedures [9]. TXA is also widely used for gynecological bleeding. A large 10-year retrospective study demonstrated both the safety and potential benefit of prophylactic TXA for postpartum hemorrhage (PPH) prevention, with 95% of peripartum patients with inherited bleeding disorders receiving TXA during delivery—most commonly administered both intrapartum and postpartum [81]. Typically given after umbilical cord clamping [82], TXA has shown an excellent safety profile, with thrombosis reported in less than 1% of women, even among those with multiple thromboembolic risk factors [81].

For hemophilia A carriers previously tested and shown to be responsive, desmopressin (DDAVP) can raise FVIII to hemostatic levels when baseline FVIII falls within the mild hemophilia range, providing a valid alternative to factor concentrates. Recent studies indicate that the new intranasal desmopressin formulation is non-inferior to earlier intranasal products and to subcutaneous desmopressin in patients with mild hemophilia A, expanding self-administration options [83]. In the peripartum period, desmopressin may be used as primary therapy for mild deficiency or as an adjunct alongside factor concentrates or TXA [80,81]. Its use requires careful monitoring for hyponatremia, a notable safety concern [80].

Despite its widespread use, a standardized definition of DDAVP responsiveness is lacking, and the biological mechanisms underlying highly variable individual responses remain unclear. In hemophilia A, generally, baseline FVIII:C appears to influence response, although the association is only borderline significant. Wide variability has been observed even among individuals carrying mutations in the same gene region [82]. A multicenter French study of 361 hemophilia A carriers found that desmopressin effectively increased FVIII levels in most participants, with 96% achieving ≥0.5 IU/mL and 79% reaching ≥0.8 IU/mL. Response varied by *F8* variant: carriers with null variants showed lower peak FVIII levels, faster clearance, and shorter duration of adequate hemostasis than those with non-null variants. Lower body weight (<35 kg) was also associated with reduced peak FVIII responses and shorter hemostatic coverage [35]. Women with FVIII deficiency and abnormal ISTH-BAT scores demonstrate markedly reduced FVIII responses to DDAVP compared with those with normal scores, which may help explain their higher rates of postsurgical bleeding [30].

Caution is required when treating patients at high thrombotic risk, including those with pre-eclampsia [82]. Although DDAVP is not strictly contraindicated, many centers prefer factor replacement therapy for hemophilia carriers at delivery to avoid hyponatremia—a side effect that may necessitate fluid restriction, which is undesirable in the setting of postpartum hypotension [82].

Traditional factor replacement therapy with recombinant FVIII and FIX concentrates remains effective for preventing and treating bleeds, including in surgical settings, although clinical trial evidence in female patients remains limited [84]. Historically, women had reduced access to factor concentrates, but recent data from the United States show increased use of replacement therapy among hemophilia A and B carriers between 2010 and 2020 compared with the previous decade. This increase was observed for both bleeding treatment and surgical prophylaxis and was more pronounced among hemophilia B carriers, likely reflecting the absence of desmopressin as an alternative therapy in hemophilia B. This trend may reflect a growing awareness of bleeding risks in female carriers and improved access to therapeutic resources in high-income settings [42].

Raso et al. emphasize that women and girls with mild FVIII or FIX deficiency should be considered mild hemophilia patients and should therefore have access to the same care and treatment strategies used for males—including prophylaxis when indicated [40]. This perspective requires careful consideration of cost-effectiveness, particularly for novel agents that may not be approved for mild disease, and underscores the importance of selecting women for prophylaxis based on individual bleeding risk rather than sex or baseline factor level alone. Novel therapies may help close the persistent care gap for males and females with non-severe hemophilia by offering prophylaxis that is not only effective but also more acceptable due to subcutaneous administration and extended dosing intervals.

A recent retrospective analysis found that, among female patients with hemophilia A, 76% were receiving prophylaxis with either factor replacement or emicizumab, with joint bleeding and pain being the most common indications [85]. Clinical trials evaluating novel hemophilia therapies frequently lack adequate inclusion of women and girls with hemophilia (WGH). The limited number of WGH treated with bispecific FVIII-mimetic antibodies have shown the expected positive outcomes, underscoring the need for inclusive trial frameworks to validate efficacy in this population [86]. Concerns remain regarding use during pregnancy. As an IgG4 monoclonal antibody, emicizumab is expected to cross the placenta, although transfer depends on maternal IgG levels, placental integrity, IgG subclass, and gestational age. Comprehensive maternal safety data remain limited [86,87,88].

Early experience with weekly subcutaneous marstacimab in seven women with non-severe hemophilia B has shown excellent bleed control, with no spontaneous breakthrough bleeds, improved quality-of-life and mobility scores, and no unexpected adverse events or thromboembolism. Notably, excellent hemostasis was achieved in a woman receiving marstacimab prophylaxis after a trauma-induced fracture, requiring only a single dose of factor concentrate for surgical prophylaxis [89]. Comparable data for concizumab in female patients are not yet available.

Use of anti-TFPI agents in women of childbearing age requires rigorous counseling regarding highly effective contraception. Beyond the risk of direct fetal exposure—since anti-TFPI agents are IgG1 monoclonal antibodies capable of crossing the placenta—there is mechanistic concern for teratogenicity related to TFPI pathway inhibition, particularly due to potential disruption of uterine and placental vascularization [90].

Fitusiran, a small interfering RNA that suppresses hepatic antithrombin production, has recently been approved for severe or moderately severe hemophilia in some countries. Its use in women could be attractive due to the markedly reduced treatment burden (administration once every two months), potentially facilitating transition to prophylaxis for women currently treated on demand. However, concerns mirror those of other novel agents, including potential teratogenicity, thrombotic risk, and safety in populations already predisposed to thrombosis [91].

Overall, systematic inclusion of women in clinical trials evaluating novel hemophilia therapies is urgently needed to ensure evidence-based treatment strategies and equitable access to emerging therapeutics.

## 9. Psychological Challenges

Women and girls with hemophilia (WGH), as well as hemophilia carriers, in addition to bleeding-related complications, experience substantial psychological and emotional challenges affecting mental health, social functioning, and overall quality of life. Women who are caregivers of affected male patients are not exempt from these challenges.

### 9.1. Women as Carriers or Affected Subjects

A major contributor to the psychological burden experienced by women with hemophilia is the persistent lack of recognition that they face substantial psychosocial challenges. Unlike individuals with cancer, perinatal complications, or other chronic illnesses—where mental health screening and support are increasingly integrated into routine care—women with hemophilia rarely receive formal psychological or psychosocial evaluation. Clinical management remains centered almost exclusively on hemostasis, while the emotional, social, and occupational consequences of living with a chronic bleeding disorder are frequently overlooked.

Women with hemophilia often experience significant emotional distress driven by uncertainty regarding their disease course and bleeding risk. This burden is compounded by the under-recognition and frequent delays in diagnosis that characterize their clinical experience. Many women are not identified as affected until adulthood, and some remain undiagnosed for years despite substantial bleeding symptoms [27]. These delays create prolonged periods during which symptoms are misinterpreted or attributed to unrelated causes, fostering anxiety, frustration, and diminished trust in the healthcare system.

The unpredictable nature of bleeding manifestations further heightens psychological strain. Because factor levels do not reliably correlate with bleeding severity in women [27], affected individuals must live with ongoing uncertainty about the timing and likelihood of serious bleeding events—including heavy menstrual bleeding, postpartum hemorrhage, and joint bleeds. This unpredictability imposes a continuous psychological load that extends well beyond isolated episodes.

For decades, women were socially and medically marginalized, viewed primarily as genetic transmitters rather than individuals with clinical symptoms [31]. This historical misconception has had lasting psychological effects, contributing to internalized stigma and reduced confidence in advocating for their own healthcare needs.

Women with hemophilia also face unique psychosocial challenges related to genetic transmission, particularly during family planning. The possibility of having a male child with severe hemophilia necessitates careful genetic counseling and complex reproductive decision-making. These considerations introduce emotional and ethical dimensions that differ markedly from typical obstetric care. The need for invasive prenatal diagnostic procedures and uncertainty surrounding fetal health further heighten anxiety during pregnancy [57].

Heavy menstrual bleeding significantly interferes with daily life, work responsibilities, and school participation. Many women report difficulty maintaining employment or continuing education due to bleeding-related complications [92]. These challenges extend beyond productivity: women often struggle with consistent attendance, managing bleeding episodes in professional environments, and coping with limited understanding or support from employers, contributing to feelings of stigma or discrimination.

Chronic bleeding frequently leads to iron deficiency anemia, which carries its own psychological consequences [27]. Fatigue, reduced stamina, and diminished daily functioning negatively affect mood and emotional well-being, creating a cycle in which reduced activity and persistent symptoms increase vulnerability to anxiety and depression.

Many women describe emotional exhaustion, isolation, helplessness, and frustration when seeking care from providers unfamiliar with hemophilia. The need to repeatedly educate clinicians and advocate for themselves—even in vulnerable situations—creates a sustained psychological burden. This “expert role” can adversely affect overall well-being and may contribute to poorer health outcomes [93].

Women with hemophilia and carriers exhibit higher rates of depression and anxiety, with greater use of related medications than controls, even among those with milder disease [94]. Compared with women with other bleeding disorders such as von Willebrand disease, quality-of-life assessments show significantly worse psychological scores, particularly for pain and anxiety/depression. Approximately 29% report bleeding symptoms, contributing to both physical and emotional burden [95].

### 9.2. Women as Caregivers of Males Affected by Hemophilia

Women with hemophilia and carriers frequently balance their own bleeding symptoms with substantial caregiving responsibilities—as mothers, sisters, daughters, and partners of affected individuals. These overlapping roles, combined with treatment challenges and pregnancy-related bleeding risks, create unique psychological pressures that warrant targeted support and dedicated resources. Even when aware of their carrier status, many women report experiencing sadness and grief upon the diagnosis of an affected son [96]. Early education for prospective mothers is essential to prevent negative emotional influences on reproductive decision-making and to support partners who may have a limited understanding of hemophilia and its implications [97]. Genetic counseling, ideally initiated before pregnancy, remains a cornerstone of informed reproductive planning [98].

Although not specific to hemophilia, research on caregivers of individuals with chronic illnesses consistently demonstrates a substantial emotional burden, including heightened anxiety, depression, and reduced quality of life among spouse or partner caregivers [99]. These findings underscore the considerable psychosocial strain borne by caregivers—particularly women—when supporting a partner with a long-term health condition. Studies focused specifically on hemophilia caregivers confirm this burden: caregivers report high levels of emotional distress, stress, and adverse health effects, with strain further amplified in families with multiple affected children [100]. Caregiving responsibilities also carry economic consequences, as caregivers of individuals with hemophilia report substantial numbers of missed workdays each year [101].

Across studies, women—whether mothers, sisters, daughters, or partners of males with hemophilia—play a central role in disease management and frequently experience significant psychosocial burden. This evidence highlights the need for dedicated resources to address the distinct psychological challenges faced by women affected by hemophilia, both in their own right and as caregivers or family members of affected males [98].

## 10. Differences Between Hemophilia A/B Carriers and Female Patients

Prospective studies specifically designed to compare hemophilia A and B carriers are required to determine whether distinct clinical management strategies are justified. A recent systematic review underscored the absence of direct comparative data on bleeding phenotypes in these two groups [102]. Because hemophilia A carriers are more frequently encountered, most available studies focus on this population, leaving potential differences in bleeding phenotype, factor level–bleeding associations, and pregnancy-related complications insufficiently defined [51]. Current evidence suggests that hemophilia A carriers exhibit higher bleeding scores than hemophilia B carriers despite comparable factor levels [103,104]. This observation may be influenced by methodological limitations, including imperfect sensitivity of bleeding assessment tools and factor assays, small sample sizes—particularly for hemophilia B—and uncharacterized biological modifiers such as genetic and hormonal factors [34,43,103].

Hemophilia B carriers display substantial phenotypic heterogeneity, with wide variability in factor IX activity and poor correlations between factor levels and bleeding severity; even mildly reduced levels (0.41–0.60 IU/mL) have been associated with clinically relevant bleeding [27]. Across carriers, menorrhagia and postpartum hemorrhage are the most frequently reported bleeding manifestations, although their relative prevalence and severity in hemophilia A versus hemophilia B carriers remain inadequately characterized [95].

## 11. Barriers to Care

Despite growing awareness, women with inherited bleeding disorders continue to face substantial obstacles in accessing appropriate care, yet these barriers remain insufficiently characterized. Challenges arise at multiple levels—individual, provider, healthcare system, and society—and collectively contribute to the persistent underdiagnosis of women with inherited bleeding disorders, a problem that may affect up to 1% of the female population [105].

Underdiagnosis and diagnostic delay represent the most fundamental barriers. The median interval between symptom onset and confirmed diagnosis can exceed ten years, particularly when heavy menstrual bleeding is the primary presenting symptom—a complaint frequently normalized or attributed to non-hematologic causes [16]. Limited awareness among healthcare providers, including primary care clinicians and especially gynecologists, contributes to symptom dismissal and missed opportunities for referral to hematology specialists. Many clinicians remain unaware that women with mild or even asymptomatic bleeding disorders are at risk of significant hemorrhagic complications, particularly during pregnancy and childbirth [6].

Qualitative evidence illustrates the lived consequences of these gaps. In a recent Canadian study involving 15 women with inherited bleeding disorders, participants reported that their condition was often poorly understood by healthcare professionals outside hemophilia treatment centers (HTCs). As a result, they frequently needed to self-explain, self-research, or even self-manage their symptoms, contributing to feelings of vulnerability and discomfort. Many described not being heard or believed, with symptom dismissal leading to strained interactions with clinicians. Participants reported limited or delayed access to appropriate treatment and insufficient clarity regarding care plans. Self-advocacy was described as essential for navigating the healthcare system, yet also “frustrating” and “unfair” [106].

Terminology also plays a role. The continued use of the outdated term *carrier* creates psychological and practical barriers, discouraging affected women from seeking care or advocating for their health needs [105]. Geographic access further constrains care quality: individuals living more than 100 miles from an HTC experience substantially higher annualized bleeding rates than those living within 50 miles [107]. Women in rural regions face compounded disadvantages, as do those in resource-limited countries where access to specialized diagnostic testing and treatment products remains severely restricted [108]. Additional barriers include insurance and language limitations, with 19.4% of patients reporting insurance challenges and 1.7% reporting language barriers to accessing HTC services [109].

Taken together, these findings underscore the urgent need for systematic efforts to identify and address the multilevel barriers that limit access to care for women with hemophilia. Improving diagnostic pathways, enhancing provider education, modernizing terminology, and expanding access to specialized services are essential steps toward equitable care for this historically underserved population.

## 12. Conclusions and Perspectives

Diagnosis and management of inherited bleeding disorders in women continue to present distinct challenges, frequently resulting in delayed identification, suboptimal treatment, and reduced quality of life. The European principles for comprehensive care (EAHAD/EHC) emphasize the need for coordinated, multidisciplinary management to address the longstanding systemic gap between the care provided to men and that provided to women with hemophilia. These principles call for structured, gender-responsive care pathways that extend beyond systematic screening and anticipated diagnosis to encompass holistic clinical, reproductive, musculoskeletal, and psychosocial support [110].

**Figure 4:**
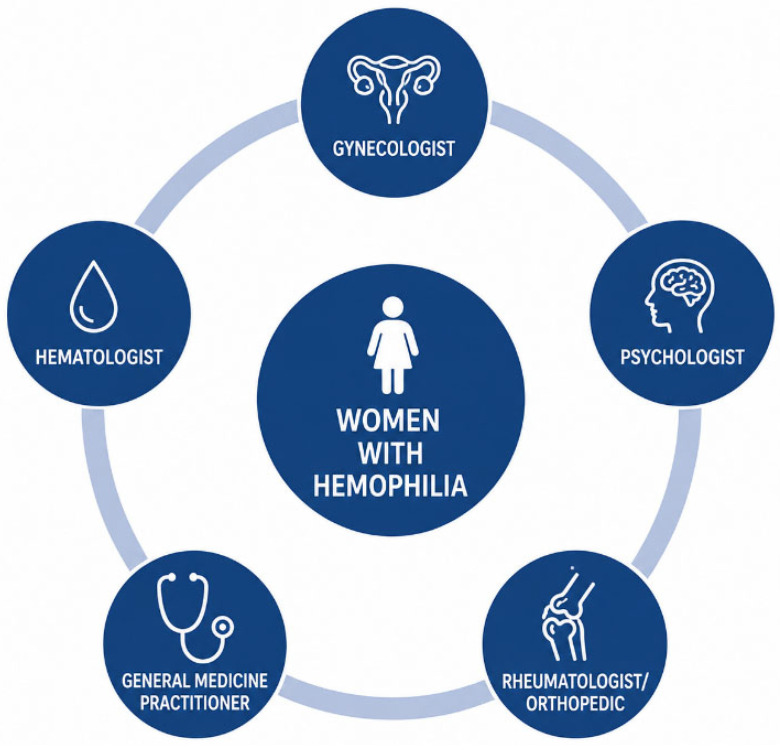
Multidisciplinary team for improving diagnosis and care for women with hemophilia.

In the authors’ personal view, a minimum number of essential specialists should be included in the multidisciplinary team at the service of female carriers and patients with hemophilia (Figure 4). This team should include hematologists, gynecologists, obstetricians, genetic counselors, psychologists or mental health professionals, physiatrists/physiotherapists, and primary care clinicians, with additional specialists involved as needed. Such coordinated expertise is critical to provide timely diagnosis, individualized bleeding management, reproductive counseling, musculoskeletal assessment, and psychosocial support—domains that remain fragmented or insufficiently addressed in current clinical practice.

Several medical centers in the United States have established multidisciplinary clinics that integrate hematology and obstetrics/gynecology services, providing an important foundation for more equitable and comprehensive care for women and girls with inherited bleeding disorders [111]. These models demonstrate the value of coordinated expertise across specialties, particularly for conditions—such as hemophilia in women—where bleeding manifestations intersect with reproductive health, musculoskeletal complications, and psychosocial needs.

A fully developed multidisciplinary approach must also incorporate mental health professionals and structured psychosocial support. Evidence from other chronic conditions affecting women’s health and reproductive outcomes [112] shows that psychological care—including counseling, cognitive–behavioral therapy, and peer support groups—improves coping, treatment engagement, and overall quality of life. Integrating these services into routine hemophilia care is essential to address the emotional burden associated with diagnostic delays, chronic bleeding, reproductive decision-making, and caregiving responsibilities. Such support not only enhances well-being but also strengthens adherence to treatment plans and fosters long-term resilience in women living with hemophilia.

**Figure 5:**
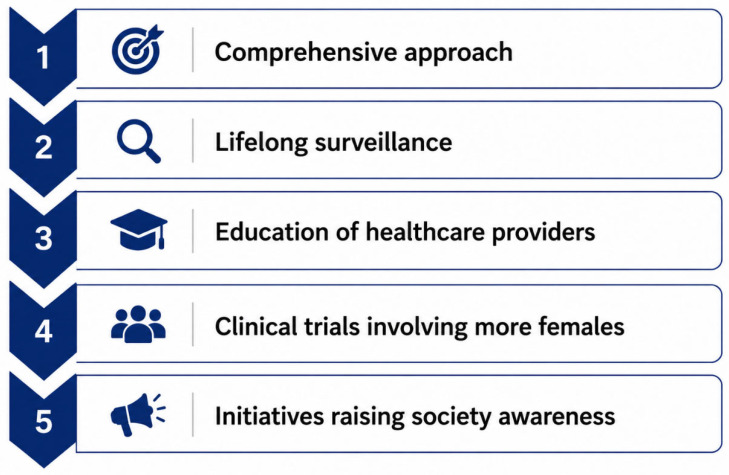
Strategies for better management of carriers and women with hemophilia.

Other measures useful for meaningful changes in the clinical management of WGBD include (Figure 5):

- A more comprehensive approach incorporating the assessment of risk factors specific to bleeding disorders, including not simply bleeding history and current factor levels but also iron status and hormonal factors;
- After referral and diagnosis, lifelong surveillance of female health, particularly focused on joint status;
- Education of healthcare providers during medical school, definitively correcting the misconception that the disorder affects only men;
- More inclusive clinical research, involving women and girls, particularly focused on specific female bleeding patterns and responses to treatment [113];
- The possible application of machine learning and artificial intelligence to bleeding risk prediction in hemophilia carriers, joint imaging diagnosis, and research [114];
- Last but not the least, an increasing number of initiatives raising social awareness about the phenomenon.

To address all the existing gaps in the management of WGBD, the Foundation for Women and Girls+ with Blood Disorders developed the “*WGBD Clinic of Excellence Model*”, a transformative framework for delivering care involving more than 70 clinics across the United States, along with 1 international site in Utrecht, the Netherlands [111].

## Data Availability

No new data were created or analyzed in this study. Data sharing is not applicable to this article.
